# An autopsy-proven case of Corticobasal degeneration heralded by Pontine infarction

**DOI:** 10.1186/s12883-021-02178-9

**Published:** 2021-04-06

**Authors:** Dallah Yoo, Sung-Hye Park, Sungwook Yu, Tae-Beom Ahn

**Affiliations:** 1 Department of Neurology, Kyung Hee University Hospital, Kyung Hee University College of Medicine, 23 Kyungheedae-ro, Dongdaemun-gu, Seoul, 02447 Republic of Korea; 2 Department of Pathology, Seoul National University Hospital, Seoul National University College of Medicine, Seoul, Republic of Korea; 3 Department of Neurology, Korea University Hospital, Korea University College of Medicine, Seoul, South Korea

**Keywords:** Corticobasal degeneration, Stroke, Autopsy, Case report

## Abstract

**Background:**

Neurodegenerative disorders are characterized by insidious progression with poorly-delineated long latent period. Antecedent clinical insult could rarely unmask latent neurodegenerative disorders. Here, we report an autopsy-proven case of corticobasal degeneration which was preceded by a lacunar infarction.

**Case presentation:**

A 58-year-old man presented with acute ataxia associated with a lacunar infarction in the right paramedian pons. His ataxia persisted with additional progressive gait difficulty and left arm clumsiness. Six months later, a follow-up neurological examination showed asymmetrical bradykinesia, apraxia, dystonic posturing, postural instability, and mild ataxia of the left limbs. Cognitive examination revealed frontal executive dysfunction and visuospatial difficulties. Dopamine transporter imaging scan demonstrated bilateral reduced uptakes in mid-to-posterior putamen, more prominent on the right side. Levodopa-unresponsive parkinsonism, asymmetric limb dystonia, and ideomotor apraxia became more conspicuous, while limb ataxia gradually vanished. The patient became unable to walk without assistance after 1 year, and died 4 years after the symptom onset. Autopsy findings showed frontoparietal cortical atrophy, ballooned neurons, and phosphorylated tau-positive astrocytic plaques and neuropil threads with gliosis and neuronal loss, confirming the corticobasal degeneration.

**Conclusions:**

The case illustrates that precedent clinical events such as stroke might tip a patient with subclinical CBS into overt clinical manifestations.

**Supplementary Information:**

The online version contains supplementary material available at 10.1186/s12883-021-02178-9.

## Background

Corticobasal degeneration (CBD) is a pathologic entity characterized by widespread tau-immunoreactive depositions in neurons and glia with a specific topographical distribution [1]. The clinical presentation of CBD is a variable combination of asymmetrical akinetic-rigid syndrome, ideomotor limb apraxia, and other clinical features including cortical sensory deficits, dystonic posturing, myoclonus, and cognitive impairment [2]. Although clinical phenotypes of CBD are heterogeneous in clinicopathologic studies, none has been reported clinical evolution of CBD consecutive to precedent non-neurodegenerative event [3]. Here, we report an autopsy-proven case of CBD which was preceded by a lacunar infarction.

## Case presentation

**Fig. 1:**
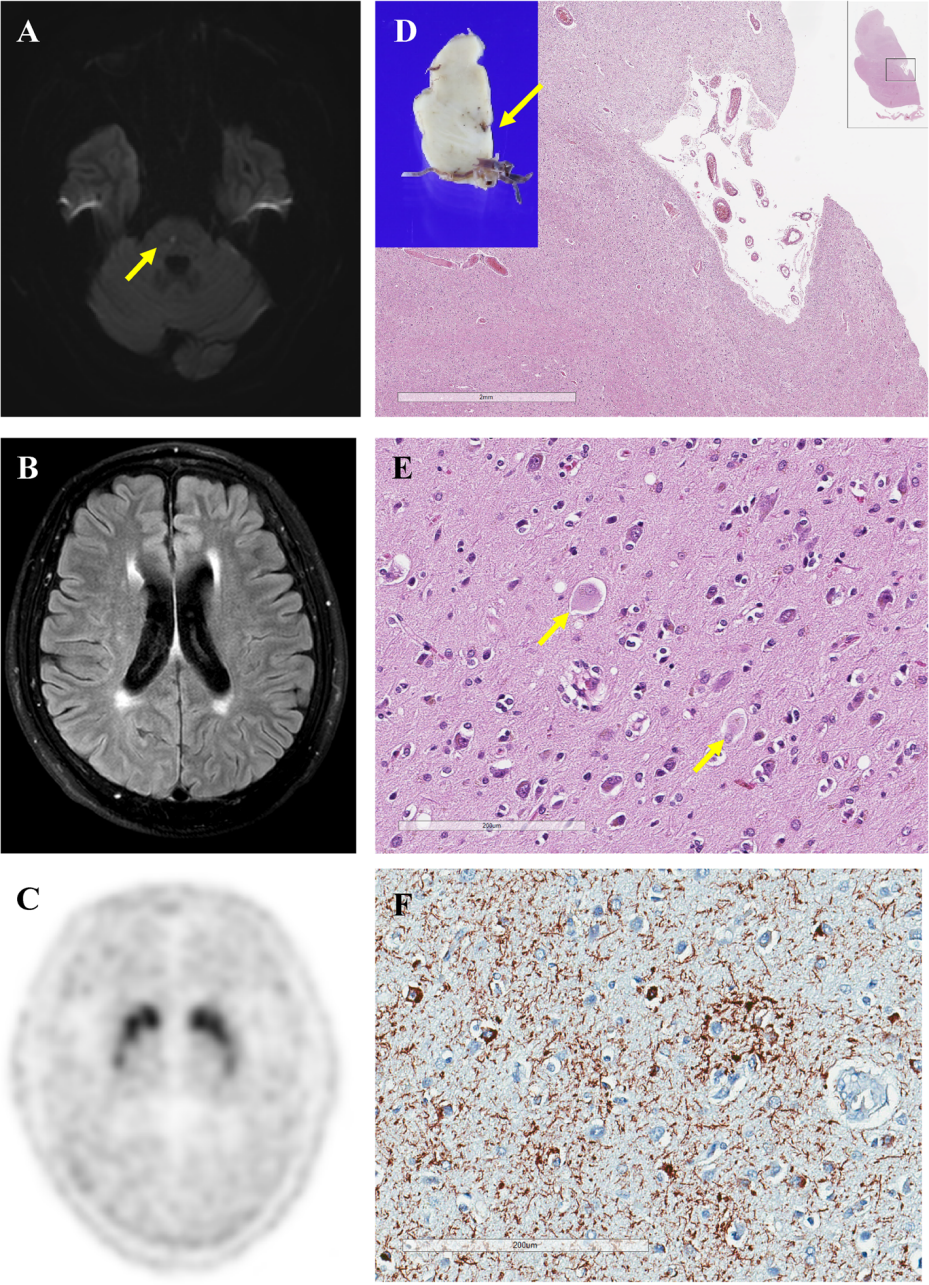
Brain imaging and autopsy findings. Focal diffusion restriction is found on the right paramedian pons on diffusion-weighted imaging (**a**, arrow). No asymmetrical cortical atrophy is found in fronto-parietal lobe on fluid attenuated inversion recovery imaging (**b**). Bilateral decreased uptakes of dopamine transporters are found on positron emission tomography using 18F-N-3-fluoropropyl-2β-carboxymethoxy-3β-(4-iodophenyl)-nortropane (**c**). Microscopic findings of the pontine lesion (arrow on macroscopic brain slice in the left upper corner) showed lacunar infarct with remaining blood vessels (Haemotoxylin and Eosin, (**h**&**e**)) (**d**). Ballooned neurons are seen in the neocortex (arrow, **h**&**e**) (**e**) Antiphosphorylated tau antibody is positive for neuronal cytoplasm, bunch of neuropil threads, and astrocytic plaques (AT8) (**f**)


A 58-year-old right-handed man visited our hospital because of acute development of incoordination of gait and left limbs. Neurological examination showed left limb ataxia with an acute paramedian pontine infarction in brain magnetic resonance imaging (Fig. 1a to b; diffusion-weighted and fluid-attenuated inversion recovery imaging, respectively). He was an ex-smoker and had no hypertension and diabetes mellitus. For the next six months, he was treated with antiplatelet but had complained progressive gait difficulty and aggravated dexterity of his left limbs. A follow-up neurological examination showed asymmetrical bradykinesia, apraxia, dystonic posturing, postural instability, and ataxia of the left limbs (Supplementary Video, Part A). Cognitive function was impaired with a Korean version of the Mini-Mental Status Examination score of 21/30 and a Frontal Assessment Battery score of 8/18. Positron emission tomography using ^18^F-N-3-fluoropropyl-2β-carboxymethoxy-3β-(4-iodophenyl)-nortropane showed decreased dopamine transporter bindings in both mid-to-posterior putamen, more apparent on the right side (Fig. 1c). Levodopa was ineffective up to 600 mg per day. Left-limb dystonia and apraxia became more conspicuous, while limb ataxia vanished. The patient was unable to walk without assistance one year later (Supplementary Video, Part B) and died four years after the onset.

**Additional file 1.**

On autopsy, gross examination showed cortical atrophy in the parietal and frontal lobes. Microscopic examination of the medial pontine lesion of the lacunar infarct showed tissue loss with remnant blood vessels (Fig. 1d, arrow on the brain slice, Haemotoxylin and Eosin, (H&E)). Astrocyte plaques and coiled bodies were found in ballooned neurons, gliosis, and neuronal loss in the parietal lobe, motor cortex, cingulate gyrus, and hippocampus (Fig. 1e, h&e). Phosphorylated tau-positive neuropil threads and astrocytic plaques were found in the frontoparietal lobes, cingulate gyrus, hippocampus, basal ganglia, thalamus, brainstem, and cerebellum (Fig. 1f, AT8). There were no inclusions stained with antibodies against β-amyloid, α-synuclein, or TAR DNA binding protein 43(TDP-43).

## Discussion and conclusions

Clinical features are summarized as acute pontine infarction and later presentation of levodopa-unresponsive asymmetric parkinsonism, dystonia, and apraxia, which are compatible with the clinical diagnosis of CBD [2].

It is worth noticing the initial presence of cerebellar ataxia. To our knowledge, there was only one case with pathologically confirmed CBD with prominent cerebellar ataxia in which TDP-43 co-existed [4]. In our case, ataxia developed as a clinical manifestation of acute pontine infarction rather than a de novo contribution of CBD. The rapid evolution of CBD, provoked by the precedent infarction, replaced ataxia with dystonia.

The mechanism of CBD flare-up after cerebral infarction remains speculative. It is more likely that aberrant pathologic network associated with tau-related neurodegeneration in CBD have constructed for years before the presentation. According to the model of cortico-basal ganglia-cerebellar connectome, an acute infarction in the pons could be detrimental to subclinical aberrant network responsible for CBD, turning latent abnormalities into clinical manifestation [5]. Alternatively, it was reported that tau protein increased in the cerebrospinal fluid after acute ischemic stroke [6]. Increased burden of tau might have provided a link between the infarction and CBD.

In the case of Parkinson’s disease (PD), some clinical events such as subdural hematoma or the use of dopamine receptor blocking agents could uncover latent PD [7]. In CBD, this is the first autopsy-confirmed CBD case unmasked by precedent cerebral infarction.

## Data Availability

All data generated or analyzed during this study are included in this published article.

## Abbreviations

CBD: Corticobasal Degeneration
TDP-43: TAR DNA binding protein-43
H&E: Haemotoxylin and Eosin
PD: Parkinson’s disease
